# Antimicrobial Zn^2+^-Carboxymethyl Chitosan Cryogel for Controlled Loading and Release of Ciprofloxacin via Coordination Bonds

**DOI:** 10.3390/gels10120841

**Published:** 2024-12-20

**Authors:** Svetlana Bratskaya, Andrey Boroda, Tamara Bogomaz, Yuliya Privar, Mariya Maiorova, Daniil Malyshev, Anastasiia Shindina, Anna Skatova, Roman Goncharuk

**Affiliations:** 1 Institute of Chemistry, Far Eastern Branch of the Russian Academy of Sciences, 159, Prosp. 100-Letiya Vladivostoka, 690022 Vladivostok, Russiamilitca2@mail.ru (A.S.); 2 A.V. Zhirmunsky National Scientific Center of Marine Biology, Far Eastern Branch of Russian Academy of Sciences, 17 Palchevskogo Street, 690041 Vladivostok, Russia; borodandy@gmail.com (A.B.); daniilmalysev46669@gmail.com (D.M.); 3 School of Medicine and Life Sciences, Far Eastern Federal University, 10 Ajax Bay, Russky Island, 690922 Vladivostok, Russia; bogomaz.tt@dvfu.ru (T.B.); shindina.ad@dvfu.ru (A.S.); goncharuk.ra@dvfu.ru (R.G.)

**Keywords:** carboxymethyl chitosan, zinc, ciprofloxacin, drug release, cytotoxicity, FT-IR spectroscopy, non-covalent interactions

## Abstract

The local application of broad-spectrum antibiotics via polymeric drug delivery systems is a promising alternative to their systemic administration in wound healing, prevention and treatment of infections associated with surgical implants. However, low and poorly controlled loading efficiency and 100% burst release are common problems for the materials with weak physical interaction between antibiotics and polymeric matrices. Here, we report a new multifunctional carboxymethyl chitosan (CMC) cryogel, which efficiently prevents bacterial adhesion to the surface, kills bacteria in the solution via controlled release of ciprofloxacin (CIP), and promotes fibroblast proliferation. The suggested approach is based on CIP loading to Zn^2+^-chelated CMC cryogel via the ligand exchange reaction. We have shown that, due to the strong binding of Zn^2+^ to CMC, the antibacterial effect and toxicity to fibroblasts of CMC-Zn-CIP cryogels were mainly determined by the content of loaded CIP, which can be precisely controlled via Zn^2+^ content in cryogel. CMC cryogels containing 20 mgZn/g can be loaded with CIP amounts sufficient to completely suppress the growth of hospital strain *Klebsiella oxytoca* with MIC of 0.125 µg/mL, while maintaining a fibroblast viability at the level of 85–90%.

## 1. Introduction

Ciprofloxacin (CIP) and other fluoroquinolones were, for many years, the most often prescribed broad-spectrum antibiotics for the treatment of urinary and other infections [1,2]. Due to the concerns about growing microorganism resistance [1], the local application of antibiotics, including fluoroquinolones [3], in wound healing and the prevention and treatment of infections associated with surgical implants is considered an alternative to systemic administration. Local drug delivery can provide higher efficiency at a reduced dose [3,4,5] and, thus, decrease antibiotic toxicity and the risk of bacterial resistance development due to the prevention of biofilm formation [3].

CIP loading on hydrogels and fibers for local applications is usually implemented via passive diffusion or impregnation [6,7,8,9,10]. However, weak interactions between fluoroquinolones and polymer matrices result in low encapsulation efficiency [7,11] and fast drug release [3], which can be prevented by expensive and multistep strategies such as the covalent conjugation of drugs to polymeric carriers [3] or mixing a solution of hydrophilic polymers with water-insoluble polymeric [12] or inorganic [13] particles pre-loaded with antibiotics.

A more elegant approach to control the pharmacokinetics of fluoroquinolones is based on non-covalent interactions, e.g., “guest–host” [14] or metal chelation metal chelation [15,16]. Formation of stable complexes between metal ions and several groups of antibiotics is an efficient route to modulate their antimicrobial activity [16,17] and bioavailability [15]. The ability of chitosan and its derivatives to form stable complexes with heavy metal ions with known antimicrobial activity [18,19,20] has promoted the development of several types of multifunctional materials containing metal ions or nanoparticles [7,8,21,22], antibiotics [7], and other bioactive factors [21,22] to provide a synergetic effect for tissue remodeling and preventing infections. Although bacteria were reported to be more sensitive to Cu^2+^ compared to Zn^2+^ ions, Cu^2+^ is more toxic to fibroblasts [23], making Zn-containing materials more promising for biomedical applications.

At concentrations from 0.001 to 0.1 μgZn/mL, zinc salts and complexes increased cell proliferation in human fibroblasts (CRL2522) and keratinocytes (HaCaT) and demonstrated antimicrobial properties in a simulated nutrient-deficient environment in vitro [24]. Additionally, 3D-printable Zn^2+^/tannic acid-reinforced glycol-functionalized chitosan hydrogel was reported to accelerate the M2 polarization of macrophages through the activation of anti-inflammatory transcription factors that provided this material with robust antibiofilm and wound-healing properties [22]. Zn^2+^ ions and ciprofloxacin-encapsulated chitosan/poly(ε-caprolactone) composite nanofibers promoted wound healing via enhanced antibacterial and immunomodulatory activity [7]. Supplementing chitosan/poloxamer-based thermosensitive hydrogels with zinc gluconate/recombinant human epidermal growth factor promoted vascular remodeling and collagen deposition, facilitated fibrogenesis, and reduced the level of interleukin 6 for wound basement repair [21]. A double-syringe injection device was used for the continuous production of CMC-Zn^2+^ supramolecular hydrogel fiber, which demonstrated antimicrobial activity against *S. aureus* and *Escherichia coli*, but at relatively high Zn loadings, whose possible negative effect on fibroblasts have not been assessed [25].

CMC often demonstrates superior properties to chitosan in biomedical applications: CMC hydrogels enhance the migration and proliferation of epithelial cells and fibroblasts, stimulate the production of collagen, and regulate gene expression [26]. In some publications, antimicrobial activity was reported for CMC with different substitution patterns and molecular weights [27,28]. Recently, we developed CMC cryogels, which were shown to be highly efficient in preventing the adhesion and colonization of both *Pseudomonas fluorescence* and *Staphylococcus aureus* on surfaces, thus demonstrating strong antifouling properties [29], but the inherent ability of CMC to kill bacteria was not confirmed.

Here, we aimed to design multifunctional CMC cryogel, which would simultaneously prevent bacterial adhesion to surfaces and kill bacteria in solutions via the controlled release of CIP. The suggested approach to increase CIP content in the polymer matrix and encapsulation efficiency in comparison with passive diffusion is based on the assumption that CIP can be loaded onto CMC-Zn^2+^ chelate cryogel via a ligand exchange reaction in a similar mode to that demonstrated earlier by our group for N-(2-carboxyethyl) chitosan–Cu^2+^ chelate for water treatment applications [30]. Since, depending on the concentration, Zn^2+^ ions can either promote or suppress fibroblast proliferation [24], and CIP can cause a decrease in collagen and proteoglycan synthesis by fibroblasts at high concentrations [31], a crucial issue in the design of multifunctional CMC-Zn-CIP cryogel was to find a balance between antimicrobial activity and cytocompatibility. Thus, dose-dependent antibacterial properties against a clinically isolated strain of *Klebsiella oxytoca* and cytotoxicity to fibroblasts were investigated in vitro for Zn^2+^ and CIP. Then, antimicrobial activity and cytotoxicity to the fibroblasts were assessed for cryogels with different loadings of Zn^2+^ and CIP.

## 2. Results and Discussion

### 2.1. Fabrication of CMC-Zn and CMC-Zn-CIP Cryogels

**Scheme 1:**
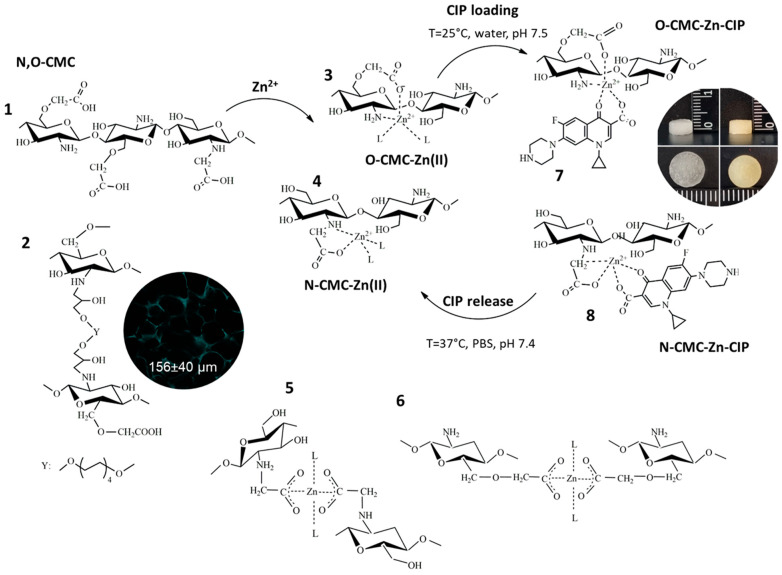
Formation of CMC-Zn-CIP cryogel via coordination bonds: structure of N,O-CMC (1), structure of CMC fragment cross-linked with BDDE and confocal microscopy image of CMC cryogel (2); possible structures of Zn^2+^ complexes with O-CMC (3) and N-CMC (4) in CMC-Zn cryogel, and corresponding structures of O-CMC-Zn-CIP (7) and N-CMC-Zn-CIP (8) in CMC-Zn-CIP cryogel. Hypothetical structures of soluble Zn^2+^-CMC complexes (5 and 6). Images in the photo are for CMC-Zn65 (left) and CMC-Zn65-CIP130 cryogel.

Polysaccharide-based hydrogels are, in general, not efficient enough to combat infections alone, but can be used as a matrix for the tunable loading and release of different antimicrobial factors [32]. In this regard, fluoroquinolones represent a promising class of antibiotics for the design of multifunctional biomaterials via coordination bonding with chitosan derivatives [25,33]. Scheme 1 shows the two-step route of CMC-Zn-CIP cryogel fabrication starting from the CMC cryogel developed earlier by our group. The original CMC cryogel covalently cross-linked using diglycidyl ether of 1,4-butandiol (BDDE) was a relatively soft material with a Young modulus of 6.9 ± 15 kPa and supermacroporous morphology with an average pore size of 156 ± 40 µm, which demonstrated high elasticity, high total swelling (5276 ± 407%), high permittivity for the cells, and low cytotoxicity [34].

**Figure 1:**
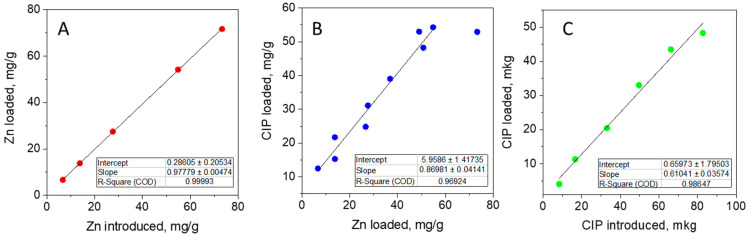
Efficiency of Zn^2+^ loading on CMC cryogel (**A**). Dependence of CIP loaded onto CMC-Zn cryogel on Zn content (**B**). Efficiency of CIP loading onto CMC cryogel with a Zn content of 30 mg/g (**C**).

At the first step, CMC-Zn cryogels with Zn contents from 2 to 85 mg/g were obtained via Zn^2+^ ion adsorption from Zn(NO_3_)_2_ solutions with concentrations optimized to provide a metal uptake efficiency of 97.7 ± 0.4% that was feasible due to the high affinity of CMC to Zn^2+^ ions (Figure 1A). Although CMC functional groups were partially involved in the covalent cross-linking reaction, the sorption capacity of the cryogel for Zn^2+^ ions remained sufficiently high (85 mg/g) in comparison with the earlier reported CMC-based materials [18,19].

**Figure 2:**
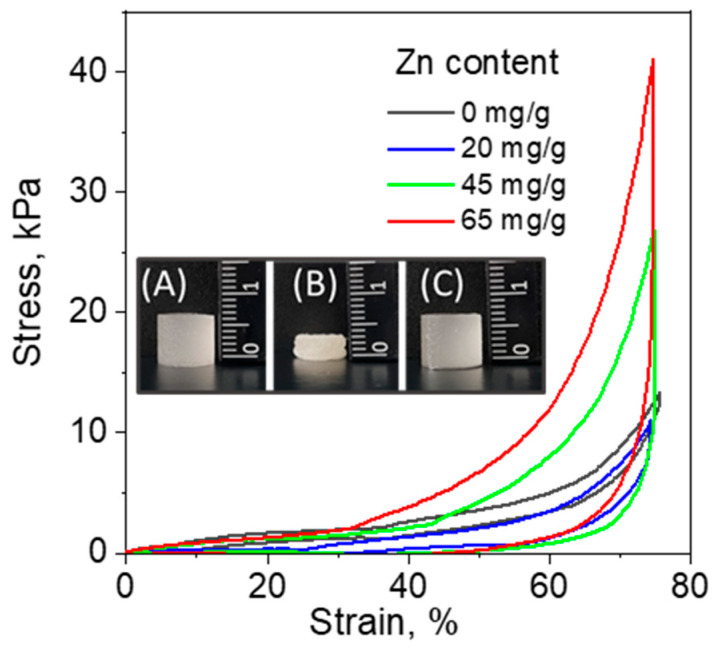
Strain–stress curves of uniaxial compression of CMC and CMC-Zn cryogels with different Zn contents. Images in the insert correspond to CMC cryogel before compression (**A**), after removal of water (**B**), and after reswelling (**C**).

The formation of coordination bonds between Zn^2+^ and functional groups of CMC resulted in a double-cross-linked network that significantly changed the mechanical properties and swelling of the original CMC cryogel. Starting from a Zn content of 20 mg/g (CMC-Zn20), we observed gradually increasing hysteresis between loading and unloading strain–stress curves (Figure 2). Observations during uniaxial compression measurements revealed that to a great extent, hysteresis originates from the inability of more rigid double-cross-linked CMC-Zn cryogels to rapidly absorb water at the unloading stage, most likely due to the changes in the capillary structure of polymer walls upon cross-linking with Zn^2+^ ions. Densification of the pore walls after Zn^2+^ loading is also evident from the difference in the strain–stress curve shape, namely from the shift of the point when stiffness starts to grow exponentially to the lower deformations (~30% for CMC-Zn65 compared to ~55% for the original CMC and CMC-Zn20). This was accompanied by a significant increase in the compressive strength, which, at the maximum deformation of 75%, reached values of 30 kPa and 41kPa for CMC-Zn45 and CMC-Zn65, respectively, while for CMC-Zn20, it remained at the level of original CMC (12 ± 2 kPa).

Earlier, we showed that metal-affine sorbents based on carboxyalkyl chitosans cross-linked with epichlorohydrin or hexamethylene diisocyanate and loaded with Cu^2+^ and Al^3+^ ions can efficiently bind CIP via ligand exchange reactions [30]. Although the stability constants of CIP-Zn^2+^ complexes are 2–4 orders of magnitude lower than those of CIP-Cu^2+^ and CIP-Al^3+^ complexes [2], one can still expect that CMC-Zn cryogels can be loaded with CIP via the formation of the mixed ligand complexes CMC-Zn^2+^-CIP.

In the second step of CMC-Zn-CIP cryogel fabrication (Scheme 1), CIP was loaded onto CMC-Zn cryogels with Zn contents ranging from 6.5 to 73 mg/g. The pH of the CIP solution was fixed to 7.5, since we showed earlier that this is the optimal pH value for CIP uptake by carboxyalkylchitosan-based metal-affine sorbents [30]. Figure 1B shows a good linear correlation (r^2^ = 0.97) between Zn^2+^ and CIP loadings that allows precise control of CIP content in the cryogels under fixed sorption conditions (CIP concentration, ionic strength, thickness of the cryogel disk, mixing rate, and contact time). The CIP loading efficiency on the CMC cryogel with a Zn content of 20 mg/g was 61 ± 3% (Figure 1C). This value was approximately two times higher in comparison to the efficiency of CIP loading on chitosan–Zn^2+^/poly(ε-caprolactone) composite nanofibers [7]. At the same time, only less than 1% (0.83 ± 0.06%) of loaded Zn^2+^ ions were released from CMC-Zn cryogels upon CIP sorption in the full range of Zn loadings shown in Figure 1A. This indicates that CIP binding to CMC-Zn^2+^ occurred via ligand exchange, and the stability of CMC-Zn^2+^ complexes was higher than that of CIP-Zn^2+^. This conclusion correlates to the literature data showing that fluoroquinolones are not effective competitors to amino acids for Zn^2+^ binding in blood plasma [35].

### 2.2. Antimicrobial Activity and Cytotoxicity

The low cytotoxicity, high permittivity for cells, and strong antifouling properties of the unmodified CMC cryogel were demonstrated earlier in 3D cell culturing applications [29,34]. Here, we have focused on the effects of Zn^2+^ and CIP loadings on the antimicrobial properties of the composite cryogel and possible dose-dependent effects of these antimicrobial factors on the viability of fibroblasts.

**Figure 3:**
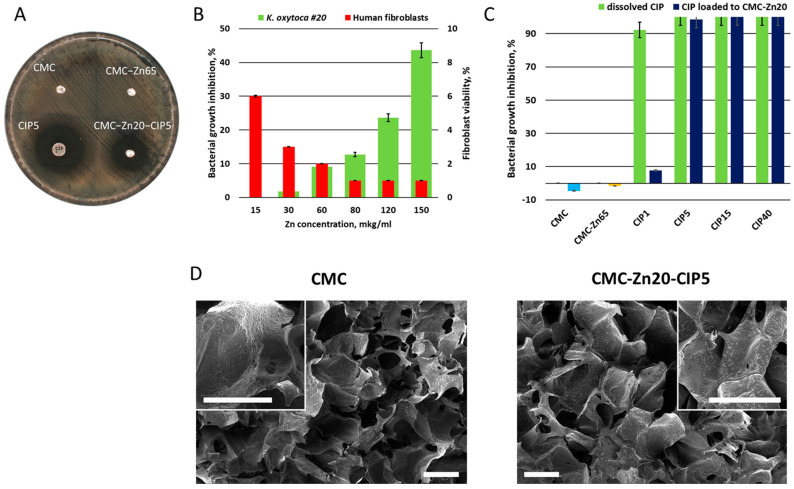
Bacterial growth inhibition on an agar plate with CMC cryogel, CMC-Zn65 (CMC with Zn content 65 mg/g), and CMC-Zn20-CIP5 (CMC with Zn content 20 mg/g loaded with 5 μg of CIP per disk) (**A**). Growth inhibition of *K. oxytoca* #20 in LB medium and viability of human fibroblasts in DMEM depending on Zn concentration (**B**). Growth inhibition of *K. oxytoca* #20 in DMEM in the presence of cryogels or dissolved CIP. CIP loadings per disk of CMC-Zn20 cryogel were equal to the doses of the dissolved CIP (**C**). SEM images of CMC and CMC-Zn20-CIP5 cryogels after cultivation of *K. oxytoca* #20 in DMEM for 3 days (scale bar 100 µm) (**D**).

First, the antimicrobial activity of CMC, CMC-Zn, and CMC-Zn-CIP cryogels against the clinical strain *K. oxytoca* #20 was investigated by the disk diffusion method. Figure 3A shows that neither CMC nor CMC-Zn cryogel with a Zn content of 65 mg/g (CMC-Zn65) inhibited bacterial growth. The earlier reported antibacterial activity of CMC-Zn supramolecular hydrogel fibers [36] is, most likely, related to the very high Zn content, which can be toxic to human cells. Figure 3B demonstrates that Zn^2+^ ions at concentrations of 15–150 µg/mL did not inhibit *K. oxytoca* growth efficiently. The notable effect of Zn^2+^ on bacteria proliferation was observed after 24 h cultivation starting from a Zn^2+^ concentration of 60 μg/mL. Bacteria growth inhibition of 55% was reached at 150 μgZn/mL. In this concentration range, the cytotoxic effect of Zn on human fibroblasts was much more significant: at 60 μgZn/mL, the fibroblast viability did not exceed 2% and decreased even more at 150 μgZn/mL.

It should be noted that the low antibacterial effect of CMC-Zn cryogels with high Zn contents could be related to the strong binding of Zn^2+^ ions by CMC. Thus, the antibacterial properties of CMC-Zn20-CIP5 cryogel evident from the disk diffusion test can be associated solely with the loaded CIP. In comparison with the commercial disk with the same CIP content (5 µg), CMC-Zn20-CIP5 exhibited a smaller inhibition zone (28 and 22 mm, respectively) that can result from the diffusion limitations for the release of CIP from the polymer matrix to agar.

To further elucidate the antimicrobial activity of CMC-Zn cryogels, a series of cryogel disks with total CIP contents of 1, 5, 15, or 40 µg were fabricated and tested in bacterial suspension in DMEM using dissolved CIP as a reference (Figure 3C). At the lowest tested CIP concentration in DMEM (0.4 µg/mL equivalent to the total CIP content of 1 μg), which is higher than the MIC for *K. oxytoca* #20 (0.125 µg/mL), bacteria growth was inhibited by 92.3% after 24 h with dissolved CIP, and only by 7.7% with CMC-Zn-CIP cryogel loaded with the same CIP content. When CIP loading on the cryogel disk was increased up to 5 µg, the efficiency of bacterial growth inhibition increased to 98%. At higher CIP contents, there was no difference in the performance of CIP dissolved and loaded on the cryogel. The bacterial growth suppression with efficiency close to 100% remained constant over 72 h.

Earlier, we showed that CMC cryogels did not suppress *S. aureus* and *P. fluorescens* growth in the suspension but efficiently prevented bacteria adhesion to the surface during 12 days of cultivation [29]. Figure 3D shows no difference in the surface morphology of cryogels, which did not prevent bacterial growth (CMC) or efficiently suppress it (CMC-Zn20-CIP5). No signs of *K. oxytoca* #20 adhesion and colonization were detected after cultivation for 72 h in the presence of unmodified CMC cryogel, despite the high turbidity of the bacterial suspension. Thus, CMC-Zn-CIP cryogels can be considered dual-function materials with antimicrobial and antifouling properties. This is advantageous since contact-killing antimicrobial surfaces often lead to the accumulation of dead bacteria and other debris, which provide nutrients and sites for other bacteria to attach and proliferate [37].

To investigate, which loadings of Zn^2+^ and CIP in CMC cryogel can be used without negative effects on human fibroblast, viability and proliferation, cytotoxicity tests were performed in a relatively small volume of DMEM, simulating the local in vivo environment around implanted material. MIC value for the tested *K. oxytoca* strain was sufficiently lower than 1 µg/mL, at which microorganisms are still considered susceptible to CIP [38]. Thus, it was important to investigate the cytotoxicity of CMC-Zn-CIP cryogels at higher loadings of Zn^2+^ and CIP than those that were found to be efficient against *K. oxytoca* (Figure 3C).

**Figure 4:**
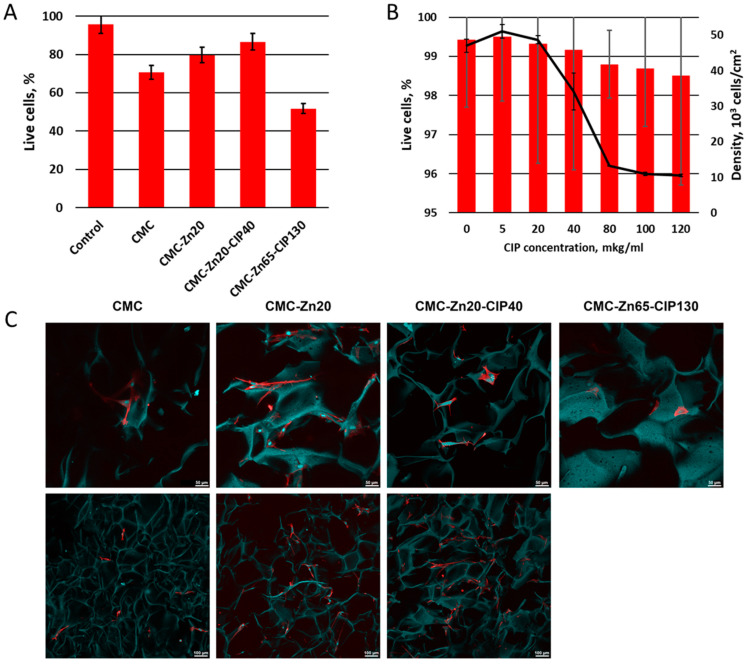
In vitro viability of human fibroblasts cultivated for 5 days in CMC, CMC-Zn, and CMC-Zn-CIP cryogels (**A**). Viability of human fibroblasts cultivated for 24 h in DMEM containing 5–120 μg/L of CIP (**B**). Confocal microscopy images of human fibroblasts cultured in CMC, CMC-Zn, and CMC-Zn-CIP cryogels, and visualized using fluorescently labeled phalloidin for staining filamentous actin (red) and DAPI for nuclear staining (cyan). Autofluorescence of the polymer (shown in cyan) can also be seen (scale bars are 50 and 100 µm in the upper and low rows, respectively) (**C**).

Figure 4A shows that after 5 days of cultivation, the viability of fibroblasts in the adhesive culture was about 95%, which is slightly higher than that of cells in the CMC cryogel—about 70%. Fibroblasts were probably more difficult to detach from the cryogel surface, especially in the case of the most viable ones, and they were dropped from the viability calculations. Nevertheless, at Zn content of 20 mg/g (CMC-Zn20), the relative positive effect on fibroblast viability in comparison to unmodified CMC cryogel was noticeable: the viability increased to 80%. Confocal microscopy images of human fibroblasts in CMC and CMC-Zn cryogels (Figure 4C) show that fibroblasts are arranged individually, without forming aggregates or clusters, indicating an even distribution of cells within the three-dimensional porous structure of cryogels. The majority of the cells exhibit a spindle-like morphology, with a less frequent polygonal shape. The visualization of the actin cytoskeleton reveals a well-developed network of actin filaments. The cells typically maintain close contact with the surface of the gel and are occasionally flattened, indicating their ability for adhesion and strong interaction with the matrix. Frequently, fibroblasts show contact areas on the walls of the cryogel’s pores, suggesting active stretching within the space between the walls. This morphology may indicate high mechanical activity, facilitating the migration of cells within the three-dimensional matrix. Visually, the general fibroblast density was slightly higher in CMC-Zn cryogel compared to CMC cryogel.

In comparison with earlier reported gelatin and gelatin-pectin cryogels doped with Zn^2+^, which resulted in 50% inhibition of human skin fibroblast growth at Zn content of 11 mg/g [39], CMC cryogels demonstrated lower cytotoxicity at higher Zn content, most likely due to the higher stability of CMC-Zn^2+^ complexes. After loading a CMC-Zn20 cryogel disk with 40 µg CIP, the fraction of viable fibroblasts increased up to 85–90% (Figure 4A). High activity and normal morphology of fibroblasts in CMC-Zn20-CIP40 cryogel were also confirmed by confocal microscopy (Figure 4C). When Zn^2+^ and CIP loadings in CMC cryogel simultaneously increased 3.25-fold (CMC-Zn65-CIP130), a cytotoxic effect on fibroblasts was evident from the reduced population of viable cells to 50–55% (Figure 4A) and changes in cell morphology (Figure 4C). The number of cells observed by confocal microscopy in CMC-Zn65-CIP130 cryogel was extremely low. The cells appeared anchored and flattened on the pore wall surface but did not exhibit the same degree of elongation as in other cryogels.

To elucidate which component of CMC-Zn65-CIP130 cryogel was responsible for increased cytotoxicity, we cultivated human skin fibroblasts in the presence of CIP for three days (Figure 4B). An increase in the CIP concentration from 5 to 120 μg/mL did not result in a noticeable decrease in fibroblast viability. However, the proliferative capacity of the cells significantly decreased at CIP concentrations >20 μg/mL. After culturing in the presence of 40 μgCIP/mL, cell density decreased from 47–50 × 10^3^ cells/cm^2^ (in control or at CIP< 20 μg/mL) to 30–35 × 10^3^ cells/cm^2^. At CIP concentrations of 80 μg/mL and higher, the density did not exceed 13 × 10^3^ cells/cm^2^. Since after incubation of CMC-Zn65-CIP130 cryogel at 37 °C in PBS for 24 h, 75–80% of CIP and less than 1% of Zn was released (see Section 2.3), one can assume a more significant contribution of CIP to the reduced cryogel cytocompatibility at high Zn^2+^ and CIP loadings. Indeed, the release of 80% of CIP from CMC-Zn65-CIP130 cryogel to DMEM resulted in a CIP concentration of ~70 μg/mL, at which a notable negative effect of CIP on fibroblast proliferation was observed (Figure 4B).

### 2.3. Binding and Release Mechanisms

The antimicrobial activity and cytotoxicity of polymeric composite materials strongly depend on the binding strength and release profiles of loaded active components. Information about the mechanism of Zn^2+^ complexation with N,O-CMC reported by different research groups is ambiguous [19,20,40]. The binding of Zn^2+^ to CMC via carboxylic groups only was suggested in [20], where higher solubility of the O,N-CMC chitosan– Zn^2+^ complex compared to the chitosan—Zn^2+^ complex was related to its enhanced antimicrobial activity against *S. aureus* and *E. coli*. The formation of soluble CMC-Zn^2+^ complexes without participation of amino groups (Scheme 1, structures 5 and 6) and of water-insoluble chelates via amino and carboxylic groups was suggested in [40].

In our case, CMC contains 46% of monomer units with primary amino groups (Table 1), so their participation in Zn^2+^ binding cannot be excluded. The fact that Zn^2+^ ions were not released upon CIP loading suggests that the stability of CMC-Zn-CIP complexes (Scheme 1, structures 7 and 8) is higher than that of Zn^2+^-CIP complexes under the experimental conditions used.

**Figure 5:**
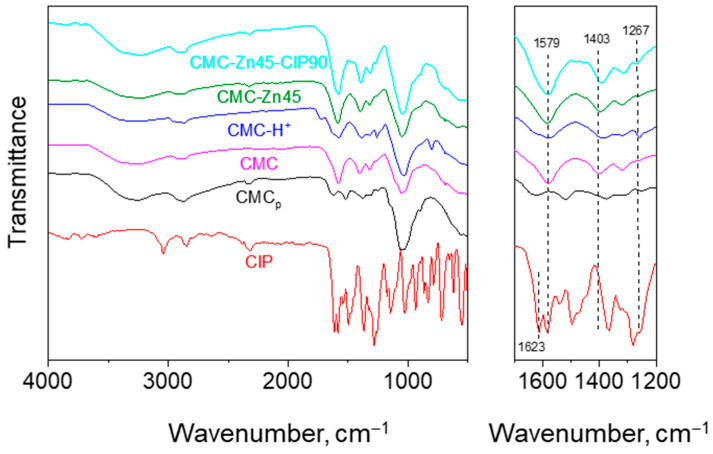
FT-IR spectra of CMC polymer (CMC_p_), dry CIP, and cryogels: CMC cryogel as synthesized (CMC), after protonation at pH 2 (CMC-H^+^), after loading with Zn^2+^ (CMC-Zn45) and Zn^2+^ and CIP (CMC-Zn45-CIP90).

The most notable difference between FT-IR spectra of CMC, CMC-Zn, and CMC-Zn-CIP cryogels was observed in the region 1200–1700 cm^−1^ (Figure 5). The stretching band of unionized and uncoordinated carboxylic groups at 1723 cm^−1^ was observed only for CMC cryogel in protonated form (CMC-H^+^). The band near 1580 cm^−1^, which can be assigned to the stretching of ionized or coordinated carboxylic groups, shifted from 1579 cm^−1^ in CMC cryogel to 1584 cm^−1^ in CMC-Zn cryogel, indicating the participation of carboxylic groups of CMC in Zn^2+^ ion binding. Although bands near 1380 cm^−1^, which shifts from 1403 cm^−1^ in CMC cryogel to 1396 cm^−1^ in CMC-Zn, can be assigned to symmetrical vibrations of carboxylic groups, in amino carboxylate–metal complexes, these bands can overlap with N–H deformation [41]. The asymmetry of the bands centered near 1580 cm^−1^ most likely results from the overlapping of bands corresponding to carboxylic and amino groups, whose binding with Zn^2+^ in chitosan resulted in the shift of the -NH band from 1624 cm^−1^ to 1628 cm^−1^ [42]. The above-discussed changes, the presence of a band at 1378 cm^−1^ in both polymers (CMC and chitosan), and the increased intensity of the band at 1257 cm^−1^ in protonated CMC cryogel, allowing its assignment to C–N stretching in amines [41], support the possibility of existence of several structures of CMC-Zn^2+^, in which amino and carboxylic groups can be coordinated to Zn^2+^ (Scheme 1, structures 3 and 4). The low antibacterial effect of CMC-Zn cryogels with high Zn contents also correlates with the strong binding of Zn^2+^ ions by CMC.

The binding of CIP to CMC-Zn cryogel via ligand exchange reactions (Scheme 1, structures 7 and 8) does not result in significant changes in the FT-IR spectra due to the numerous overlapping bands of CIP and CMC. However, the presence of CIP in CMC-Zn-CIP cryogel can be confirmed by the notable increase in band intensity at 1267 cm^−1^ and visible asymmetry of the band centered in CMC-Zn45 cryogel at 1584 cm^−1^ due to the overlap with the intensive CIP band at 1623 cm^−1^ (Figure 4).

In comparison with the earlier reported CIP encapsulation in the polymer matrices via passive diffusion [6,7,8], loading CIP on CMC-Zn cryogel via ligand exchange reaction is beneficial in terms of loading efficiency and precise control of CIP contents. Since the CIP encapsulation efficiency in both chitosan/poly(ε-caprolactone) and chitosan/Zn^2+^/poly(ε-caprolactone) fibers was below 40% and was not notably improved in the presence of Zn^2+^ [7], one can attribute the observed CIP loading enhancement to the difference in CIP binding mechanisms to CMC-Zn^2+^ and chitosan–Zn^2+^ complexes.

**Figure 6:**
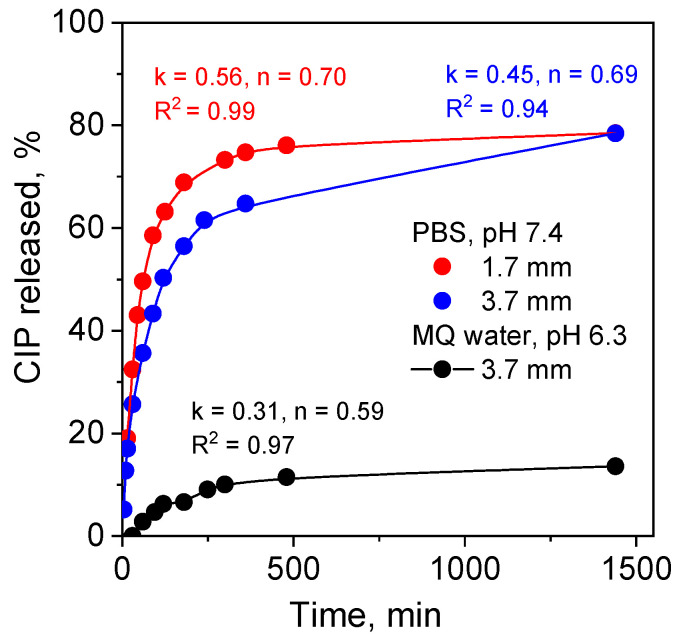
Profiles of CIP release from the disks of CMC-Zn65-CIP130 cryogels with thicknesses of 1.7 and 3.7 mm in PBS, pH 7.4 and ultrapure (MQ) water, pH 6.3, T = 37 °C.

Saturation of the polymers with CIP via passive diffusion and subsequent release of antibiotic is based on the pH dependence of CIP solubility, which is minimal at pH 7.4 and significantly increases at pH < 5.5, resulting in the burst effect of CIP release in the first 5–7 min [43]. CIP release from CMC-Zn-CIP cryogels was triggered by an increase in the ionic strength (Figure 6). Kinetic parameters of CIP release from the disks of CMC-Zn65-CIP130 cryogel in PBS at 37 °C calculated using the Korsmeyer–Peppas model gave release exponent (n) values of 0.69–0.70 and release constants of 0.45 and 0.56 (min^−1^) for the disks with thicknesses of 1.7 and 3.6 mm, respectively. The half-time of CIP release increased from 60 to 123 min when the disk thickness was doubled.

These values of the release exponent correspond to anomalous non-Fickian behavior, which can be related to the strong contribution of swelling and relaxation in the polymer matrix to the drug-release mechanism. It is not surprising, considering the high swelling degree of cryogels (>5000%) and the presence of not only free (“squeezable”) water in macropores but also water bound to the polymer. This structure assumes the existence of stagnation zones inside the swollen supermacroporous disks, where mass transfer is hindered even at high rates of external stirring, as we demonstrated earlier in the experiments with metal ion binding to cryogel [44]. Thus, one can expect that the release rate from CMC-Zn-CIP cryogels can be tuned via the modification of the porous structure and pore wall thickness.

Despite the difference in CIP release profiles depending on the disk thickness, in both cases, only 78 ± 1% of CIP was released after 72 h. The CIP fraction, which remained in the cryogel, can be assigned to the most stable mixed ligand CMC-Zn-CIP complexes, which are released only upon bioresorption of the CMC cryogel. The presence of this irreversibly bound CIP fraction would be beneficial for the prevention of biofilm formation during the tissue remodeling process, while relatively fast release within the first 24 h is essential to stop the development of possible implant-associated infection [45].

## 3. Conclusions

Polysaccharide-based hydrogels are, in general, not efficient enough to combat infections alone, but can be used as a matrix for the tunable loading and release of different antimicrobial factors. In this regard, fluoroquinolones represent a promising class of antibiotics for the design of multifunctional biomaterials via coordination bonding with chitosan derivatives and metal ions. Here we have suggested a new approach to impair early developed by our group carboxymethyl chitosan (CMC) cryogel with antimicrobial properties via formation of coordination bonds between CMC and Zn^2+^ ions followed by ciprofloxacin (CIP) loading via ligand exchange reaction CIP loading depended linearly on Zn content in cryogels, the CIP loading efficiency to CMC-Zn cryogel containing 20 mgZn/g was 61 ± 3%.

Since at certain concentrations, both Zn ^2+^ ions and CIP can be cytotoxic to fibroblasts and negatively affect the potential of the cryogels for tissue regeneration, it was crucial to find a balance between the antimicrobial activity and cytocompatibility of CMC-Zn-CIP cryogels. It has been shown that 55% inhibition of hospital strain *K oxytoca* #20 growth in suspension was achieved only at a Zn^2+^ ion concentration of 150 μg/ml, while fibroblast viability did not exceed 2% already at Zn^2+^ concentration of 60 μg/ml. Due to the high stability of the CMC-Zn^2+^ complex, cryogels with a high Zn content (85 mg/g) did not exhibit antibacterial properties and were not cytotoxic to fibroblasts. The antibacterial properties of CMC-Zn-CIP cryogels investigated by the disk diffusion method and in suspension tests were dependent on the content of loaded CIP. The cytotoxic effect of cryogels with high Zn^2+^ and CIP contents was also associated with the suppressive effect of antibiotic on fibroblast proliferation at concentrations above 20 mg/L.

It was found that cryogels with a Zn content of 20 mg/g can be loaded with ciprofloxacin in amounts sufficient to completely suppress the growth of *K. oxytoca* #20 with an MIC of 0.125 µg/mL and potentially of microorganisms susceptible to CIP (MIC ≤ 1 μg/mL), while maintaining a fibroblast viability level of 85–90%.

The relatively fast initial release of CIP (half-life time of 60–123 min in PBS at 37 °C) and slow release of 22 ± 1% of irreversibly bound CIP during cryogel bioresorption are potentially beneficial to prevent the development of implant-associated infections.

## 4. Materials and Methods

### 4.1. Materials

N,O-(carboxymethyl)chitosan (CMC) with a degree of carboxyalkyl substitution of 1.49 was purchased from BioLog Heppe GmbH (Landsberg, Germany) in the form of sodium salt. The CMC monomer composition was determined by ^1^H NMR spectroscopy (Table 1). 1,4-butanediol diglycidyl ether (BDDGE) 95% and dry ciprofloxacin (CIP) were purchased from Sigma-Aldrich(St. Louis, MO, USA). PBS tablets and CIP solution for infusion were purchased from “Paneco” Ltd. (Moscow, Russia) and “Ist-Farm” Ltd. (Ussuriysk, Russia), respectively. Other reagents were of analytical grade. Milli Q Ultrapure Water was used for the preparation of the solutions.

### 4.2. Fabrication of CMC and CMC-Zn Cryogels

A 3% CMC solution was prepared in distilled water; the resulting pH of the solution was 10.2. BDDE (0.143 g) and CMC solution (10 g) were mixed and left for 10 min under constant stirring at 25 °C. Then, the solution was placed into plastic syringes with an inner diameter of ~0.85 cm and 0.44 cm (for the disk diffusion test only) and kept in a freezer (Liebherr, Kirchdorf an der Iller, Germany) at –10 °C for 7 days. After thawing at 25 °C, the CMC cryogels were thoroughly washed with distilled water using a peristaltic pump (Ismatec, Wertheim, Germany). The CMC cryogels were cut into disks with a thickness of ~1.7 or 3.6 mm for further modification.

Disks of CMC-Zn cryogels with Zn content ranging from 2 to 85 mg/g were obtained by adsorption from Zn(NO_3_)_2_ solutions with concentrations of 0.15–4.5 mM. The precise contents of Zn in cryogels were calculated from the differences in initial and equilibrium concentrations of Zn determined by atomic absorption spectrometry (AAS) using a Shimadzu 7000 spectrometer (Shimadzu, Kyoto, Japan). The obtained CMC-Zn cryogels were thoroughly washed with distilled water and stored swollen and sealed until use. Cryogels are labeled as CMC-Zn with the content of Zn in mg/g, e.g., CMC-Zn20 means CMC cryogel with a Zn loading of 20 mg/g.

### 4.3. Ciprofloxacin Loading and Release

A stock solution (50 mL) was prepared by dissolving 4.14 mg of ciprofloxacin (CIP) in 0.2 mL of 1M HCl, followed by the addition of ultrapure water and pH adjustment to 7.5 with 1M NaOH solution. CIP was loaded onto CMC-Zn cryogels with different Zn contents from 0.25 mM or 0.05 mM CIP solutions at a solid/liquid ratio of 1:1000 and 120 rpm (Orbital shaker Biosan PSU-10i (SIA Biosan, Riga, Latvia)). To find correlations between CIP and Zn loadings, the cryogel disks with a thickness of 1.7 mm in the swollen state were used, and the contact time was fixed to 18 h. The equilibrium concentration of CIP was determined spectrophotometrically at a wavelength of 272 nm using a UV-1650PC spectrophotometer (Shimadzu, Japan). The precise contents (µg) of CIP in the cryogel disks were calculated using the difference in initial and equilibrium CIP concentrations. The CMC-Zn-CIP cryogels were labeled with the content of Zn in mg/g and total content of CIP in the disk, e.g., CMC-Zn20-CIP5 means a disk of CMC cryogel with a Zn loading of 20 mg/g and a CIP content of 5 µg.

The CIP loading efficiency to CMC-Zn20 cryogel disks (~2 mg of dry weight) was investigated at a solid/liquid ratio of 1:500 for a CIP concentration range of 0.025–0.25 mM and calculated from the slope of the dependence of CIP content loaded onto the disk vs. CIP content in solution.

CIP release from CMC-Zn65-CIP130 cryogels with thicknesses of 1.7 mm and 3.6 mm was investigated as follows: disks were immersed in 4 mL of PBS buffer (pH 7.4) and thermostated at 37 °C; CIP concentrations in the solutions were determined spectrophotometrically as described above at time points from 5 min to 24 h. The Zn concentration in PBS solution after CIP desorption was determined by AAS. The kinetics of CIP release were fitted using the Korsmeyer–Peppas model [46]:

(1) log(Mt/M∞)=log(K)+n·log(t)

where Mt and M∞ are cumulative amounts of CIP released at time t and the maximum amount released under the experimental conditions used; n is the release exponent indicating the release mechanism; K is the release rate constant.

To confirm the material balance in CIP release experiments, cryogel disks were removed from PBS after 72 h, washed with ultrapure water, and immersed in 0.01M HCl solution for complete release of CIP and Zn from the polymer matrix.

### 4.4. Mechanical Properties of Cryogels

Uniaxial compression tests at a constant speed of 0.01 mm/s were performed using a Physica MCR 301 rheometer (Anton Paar GmbH, Graz, Austria) as decribed in [47]. Loading and unloading strain–stress curves have been calculated from the measured normal force (F_N_) and gap. Compressive strength was determined at 75% strain.

### 4.5. Antibacterial Activity

Clinically isolated *Klebsiella oxytoca* #20 from the collection of the Medical Complex (Center for Laboratory Research) of the Far Eastern Federal University was grown on agar containing 5% sheep blood («Sredoff», Saint-Petersburg, Russia) for 24 h, suspended in the liquid medium Miller’s LB Broth (MP Biomedicals, LCC, Illkirch-Graffenstaden, France) and grown overnight. Then, overnight culture was diluted with LB Broth to adjust turbidity to 0.5 McFarland standard using a MicroScan Turbidity Meter (Siemens AG, Munich, Germany).

To investigate the antibacterial properties of the cryogels, disks with a thickness of 1.5 mm and diameter of 5 mm were placed on the surface of agar plates with spread bacterial suspension. A Bio-Rad antimicrobial susceptibility disk with a CIP content of 5 µg was used as a reference. The inverted plates were incubated at 37 °C for 24 h. Images of the inhibition zones were captured using an ADAGIO BioRad analyzer (Marnes-la-Coquette, France); the diameters of the inhibition zones and MIC for ciprofloxacin were calculated using the built-in software. Quality control testing of CIP disks was performed every 7 days using *Escherichia coli* ATCC 25922 and *Staphylococcus aureus* ATCC 29213 strains.

Quantitative analysis of bacteria sensitivity to Zn^2+^ ions and soluble form of CIP was performed in LB Broth, to CIP bound to cryogels in DMEM (#D5648, Sigma-Aldrich, St. Louis, MO, USA). In total, 25 µL of bacterial suspension (turbidity of 0.5 MFU) was added to the test tubes with 2.5 mL of liquid medium containing (i) zinc nitrate at concentrations of 15–150 µgZn/mL; (ii) 1, 5, 15, and 40 µg of CIP; (iii) cryogel disks (2.5 mg of dry weight)—unmodified CMC, CMC with Zn content 65 mg/g (CMC-Zn65), CMC with Zn content 20 mg/g (CMC-Zn20) loaded with 1, 5, 15, and 40 µg of CIP per disk. Additionally, 25 µL of bacterial suspension in 2.5 mL of medium without additives was used as a control (T_control_). Test tubes were incubated at 37 °C for 24 h under aerobic conditions, then the turbidity was measured using a MicroScan Turbidity Meter. Turbidity of medium (T_0_) was used as a background signal.

Bacterial growth inhibition (I) was calculated using the following equation:

(2) $$I=(\left(T_{\mathrm{sample}}-T_{0}\right)/\left(T_{\mathrm{control}}-T_{0}\right))\times 100\%$$

where T_sample_, T_control_, and T_0_ are the turbidity of the sample, control, and background, respectively.

### 4.6. Cytotoxicity

#### 4.6.1. Isolation and Cultivation of Human Dermal Fibroblasts (HDFs)

A skin punch biopsy 3 mm wide and 3 mm deep from the shoulder area was conducted at the Medical Center of Far Eastern Federal University with the voluntary consent of the donor. The skin tissue was minced and enzymatically disaggregated into cells for 90 min at +37 °C in the presence of 2 units/mL collagenase type IV (Sigma-Aldrich, St. Louis, MO, USA). The resulting cell suspension was seeded in T75 flasks (TPP, Trasadingen, Switzerland) in Dulbecco’s modified Eagle’s medium (DMEM, #D5648, Sigma-Aldrich) enriched with 10% (*v*/*v*) fetal bovine serum (FBS, HyClone, Logan, UT, USA), 3.7 mg/mL sodium bicarbonate (Sigma-Aldrich), 1× non-essential amino acid mixture (MEM NEAA, Sigma-Aldrich), 100 units/mL penicillin (Sigma-Aldrich), and 100 µg/mL streptomycin (Sigma-Aldrich). The cells were cultured in a CO_2_ incubator at +37 °C, 5% CO_2_, and 90% relative humidity until 90% confluency was reached. Then, HDFs were detached with 0.05% (*w*/*v*) trypsin and 0.02% (*w*/*v*) EDTA solution at +37 °C for 10 min, centrifuged at 500× *g* for 5 min, resuspended in fresh medium without antibiotics, and used for cytotoxicity tests or seeding into cryogel disks.

#### 4.6.2. Cytotoxicity Tests with Zn^2+^ and CIP

HDFs were seeded in adhesive 24-well culture plates at 100 × 10^3^ cells/well in 1 mL of DMEM. Then, 0.01M Zn(NO_3_)_2_ solution or CIP solution for infusion (“Ist-Farm” Ltd., Ussuriysk, Russia) was added to the wells to final concentrations of 15, 30, 60, 80, 120, and 150 µg/mL for Zn, and 5, 20, 40, 80, 100, and 120 µg/mL for CIP.

The fibroblasts were cultured at +37 °C, 5% CO_2_ for 24 h, detached from the wells with 0.25% (*w*/*v*) trypsin–0.05% (*w*/*v*) EDTA solution at +37 °C for 20 min, triple washed with 2 mL of DPBS, and centrifuged at 500× *g* for 5 min. Their viability was assessed as described in Section 4.6.4 and Section 4.6.5.

#### 4.6.3. Cultivation of Human Dermal Fibroblasts in Cryogels

Swollen cryogel disks (dry weight equivalent of 3 mg) were put in wells of a 24-well culture plate, and washed with 5 mL of Dulbecco’s phosphate buffer saline (DPBS, Sigma-Aldrich) without Ca^2+^ and Mg^2+^, and 5 mL of DMEM without antibiotics. All liquid was aspirated from the cryogel disks. HDFs were suspended in DMEM and seeded on top of a cryogel disk at a density of 100 × 10^3^ cells/disk. The samples were cultivated in 1.5 mL of DMEM per well at +37 °C and 5% CO_2_. The medium was replaced on the 2nd and 4th days.

After 5 days of cultivation, the cryogel disks with cells were either fixed for confocal laser scanning microscopy or transferred to a new 24-well culture plate, washed twice with 1 mL of DPBS, and minced with a scalpel (for viability assay only). Cells were detached from the cryogel disks with 0.25% (*w*/*v*) trypsin–0.05% (*w*/*v*) EDTA solution at +37 °C for 20 min, triple washed with 2 mL of DPBS, filtered through a 40 µm nylon mesh, and centrifuged at 500× *g* for 5 min.

#### 4.6.4. Assessing Fibroblast Viability

Cells from a single well of a culturing plate or cryogel disk were suspended in 100 µL of DPBS with 1 µM SYTO 9 (Sigma-Aldrich) to stain only cells and not to stain cryogel fragments and 10 µM 2′,7′-dichlorodihydrofluorescein diacetate (H_2_DCFDA) (Sigma-Aldrich) to assess the activity of mitochondria (fibroblast viability). The cells were incubated in the dark at room temperature for 10 min, diluted with 100 µL of DPBS, and then flow cytometric analyses were performed using a CytoFLEX flow cytometer (Beckman-Coulter, Brea, CA, USA) and CytExpert software (version 2.6, Beckman-Coulter). The detailed analysis is given in [47].

#### 4.6.5. Confocal Laser Scanning Microscopy (CLSM)

Cryogel disks with cultured HDFs were fixed in 4% paraformaldehyde (PFA; Sigma-Aldrich) in DPBS for 60 min at +4 °C and rinsed 3 times with DPBS. The disks were incubated overnight in a solution of tetramethylrhodamine-conjugated phalloidin (Santa Cruz Biotechnology, Dallas, TX, USA) at +4 °C to detect filamentous actin; then, they were washed three times with DPBS followed by staining with 10 μg/mL 4′,6′-diamidino-2-phenylindole (DAPI, Sigma-Aldrich) to reveal the nuclei. The stained material was stored in DPBS with ProClin preservative (Sigma-Aldrich) at +4 °C in the dark. The cryogel section was placed in Vectashield medium in a Petri dish with a thin (0.17 mm) glass bottom immediately before the microscopy analysis. The fibroblast morphology was studied under an LSM 800 confocal laser scanning microscope (Carl Zeiss, Oberkochen, Germany). To visualize the interaction of cells with the matrix, several fields of each sample were scanned at a magnification of 10× and at a magnification of 20×. Z-projections and multichannel images were assembled using ImageJ software v. 1.54k (NIH, Bethesda, MD, USA).

### 4.7. Scanning Electron Microscopy

Cryogel disks with cultured bacteria were fixed with 2.5% glutaraldehyde (EMS, USA) solution in 0.1 M cacodylate buffer (Sigma-Aldrich), passed through increasing concentrations of ethanol (15, 30, 50, 70, and 96%) with a 30 min incubation at each step, dried at the critical point, and coated with chromium. The analysis was conducted under a scanning electron microscope, Sigma VP (Carl Zeiss, Germany).

## Figures and Tables

**Table 1:** Monomer composition of carboxymethyl chitosan (CMC).

DA ^1^	DS_tot_^2^	N-DS ^3^	O-DS ^4^	Monomer Composition			
				NH_2_	NHR	NR_2_	NHCOCH_3_
0.25	1.49	0.29	1.20	0.46	0.29	0	0.25

^1^—degree of acetylation; ^2^—degree of carboxyalkyl substitution (total); ^3^—degree of N-carboxyalkyl substitution; ^4^—degree of O-carboxyalkyl substitution.

## Data Availability

The original contributions presented in this study are included in the article. Further inquiries can be directed to the corresponding author.
